# No-ozone cold plasma can kill oral pathogenic microbes in H_2_O_2_-dependent and independent manner

**DOI:** 10.1038/s41598-022-11665-z

**Published:** 2022-05-09

**Authors:** Nam-Sook Park, Se-Eun Yun, Hyun-Young Lee, Hae June Lee, Jeong-Hae Choi, Gyoo-Cheon Kim

**Affiliations:** 1 Department of Research and Development, FEAGLE Corporations, 70-6, Jeungsan-ro, Mulgeum-eup, Yangsan-si, Gyeongsangnam-do 50614 South Korea; 2 Department of Oral Anatomy and Cell Biology, School of Dentistry, Pusan National University, Busan, South Korea; 3 Department of Electrical Engineering, Pusan National University, Busan, South Korea; 4 Department of Anatomy and Cell Biology, School of Dentistry, Yangsan Campus of Pusan National University, Beomeo-ri, Mulgeum-eup, Yangsan-si, Gyeongsangnam 626-870 South Korea

**Keywords:** Antimicrobials, Oral microbiology, Biomedical engineering, Plasma physics, Microbiology

## Abstract

To apply the sterilisation effect of low-temperature plasma to the oral cavity, the issue of ozone from plasma must be addressed. In this study, a new technology for generating cold plasma with almost no ozone is developed and is named Nozone (no-ozone) Cold Plasma (NCP) technology. The antimicrobial efficacy of the NCP against four oral pathogens is tested, and its specific mechanism is elucidated. The treatment of NCP on oral pathogenic microbes on a solid medium generated a growth inhibition zone. When NCP is applied to oral pathogens in a liquid medium, the growth of microbes decreased by more than 10^5^ colony forming units, and the bactericidal effect of NCP remained after the installation of dental tips. The bactericidal effect of NCP in the liquid medium is due to the increase in hydrogen peroxide levels in the medium. However, the bactericidal effect of NCP in the solid medium depends on the charged elements of the NCP. Furthermore, the surface bactericidal efficiency of the dental-tip-installed NCP is proportional to the pore size of the tips and inversely proportional to the length of the tips. Overall, we expect this NCP device to be widely used in dentistry in the near future.

## Introduction

More than 700 kinds of microorganisms exist in the human oral cavity and form one of the most complex microbial communities in the human body^1^. Although most of these microorganisms exist as harmless commensal flora, some have been reported to be directly or indirectly related to the occurrence of dental diseases such as dental caries, periodontal disease, and oral cancer^2–4^. For example, *Streptococcus mutans* (*S. mutans*) adheres to the dental surface and generates acid on the tooth surface via carbohydrates, thereby generating dental caries by decaying the teeth^5^. Meanwhile, *Porphyromonas gingivalis* (*P. gingivalis*) was detected in the subgingival plaque of approximately 86% of patients with periodontitis, and it is well known as one of the major etiological agents that induce inflammatory reactions in periodontal diseases^6,7^. *Enterococcus faecalis* (*E. faecalis*) is an anaerobic gram-positive coccus, which is typically a normal commensal flora but widely regarded as a representative endodontic pathogen that is present in approximately 90% of post-endodontic therapy pain and infection cases^8,9^. *Candida albicans* (*C. albicans*) is a harmless fungus that is observed with a probability of 80% or higher in the oral cavity of healthy people; however, it can cause oral candidosis owing to its abnormal growth under certain conditions^10^. Therefore, for the successful treatment of pathogen-driven dental diseases such as dental caries and periodontitis, the effective removal of these pathogenic microorganisms from the lesion is essential^11^. In most dental procedures, dentists typically remove the infected tissues physically and then clean out residual pathogens using antibiotics and antifungal agents to minimise secondary infection. However, some oral pathogens are resistant to certain drugs^12^, and side effects caused by these drugs have been reported^13^. Therefore, a new option that can kill pathogens more effectively while minimising the use of these drugs is necessitated.

Recently, the use of low-temperature plasma for medical purposes has been investigated extensively^14–16^. Research pertaining to the medicinal effects of low-temperature plasma, such as sterilisation^17,18^, anti-cancer^19,20^, anti-inflammation^21^, transdermal drug delivery^22,23^, and tissue regeneration^24,25^ are being actively conducted by numerous researchers, and based on the results, various medical low-temperature plasma generating devices are being developed. In particular, the strong sterilisation effect of low-temperature plasma has been investigated, and medical sterilisers using plasma have been released^26^, additionally, various types of plasma wound treatment devices that can promote wound healing through skin surface sterilisation are being developed^27,28^. Various studies regarding the dental application of low-temperature plasma are being actively pursued^29,30^. To date, numerous researchers have reported that low-temperature plasma can be adopted for tooth whitening^31^, fluoride coating^32^, enhancement of dental adhesive efficacy^33–36^, and peri-implantitis^37^. In particular, the sterilisation effect of low-temperature plasma against various oral microbes such as *S. mutans*^18^, *E. faecalis*^38^, *P. gingivalis*^39^ and *C. albicans*^40^ have been reported. Nevertheless, to use low-temperature plasma for dental treatment, two significant issues must be addressed. One of the issues pertain to ozone. Ozone can be additionally generated during low-temperature plasma generation; ozone generation must be minimised because it can trigger severe damages to the respiratory system. The other issue pertains to temperature. To discriminate dental plasma technology from previously adopted dental laser, the temperature of the plasma must be maintained below 37 °C to avoid thermal damage. Plasma microbial killing effects are caused by the combination of several elements of the plasma, such as ions, electrons, reactive oxygen and nitrogen species, heat, UVs, and ozone^41^. Hence, plasma sterilisation effects can vary depending on the plasma generation method used. Therefore, even if a plasma generation technology that minimises ozone and heat generation is developed for dental applications, one must verify whether it can effectively sterilise various oral pathogenic microorganisms. Additionally, a method that can effectively deliver the sterilisation effect of low-temperature plasma to complex-structured oral tissues must be developed.

In this study, we developed a novel dental plasma device that generates a specific type of plasma that minimises the ozone level at a low temperature (under 30 °C); we named this plasma the ‘no-ozone cold plasma (NCP)’. As the NCP device developed in this study was designed to mount dental tips that have been used for various dental procedures, the NCP’s antimicrobial effects against four types of oral microbes (*S. mutans, E. faecalis, P. gingivalis*, and *C. albicans*) were investigated with or without the mounting of dental tips. In addition, by conducting various studies to investigate the mechanism of bactericidal action of NCP, the possibility of dental application of NCP was analysed.

## Results

### Development of low-ozone cold plasma system for dental application

**Figure 1:**
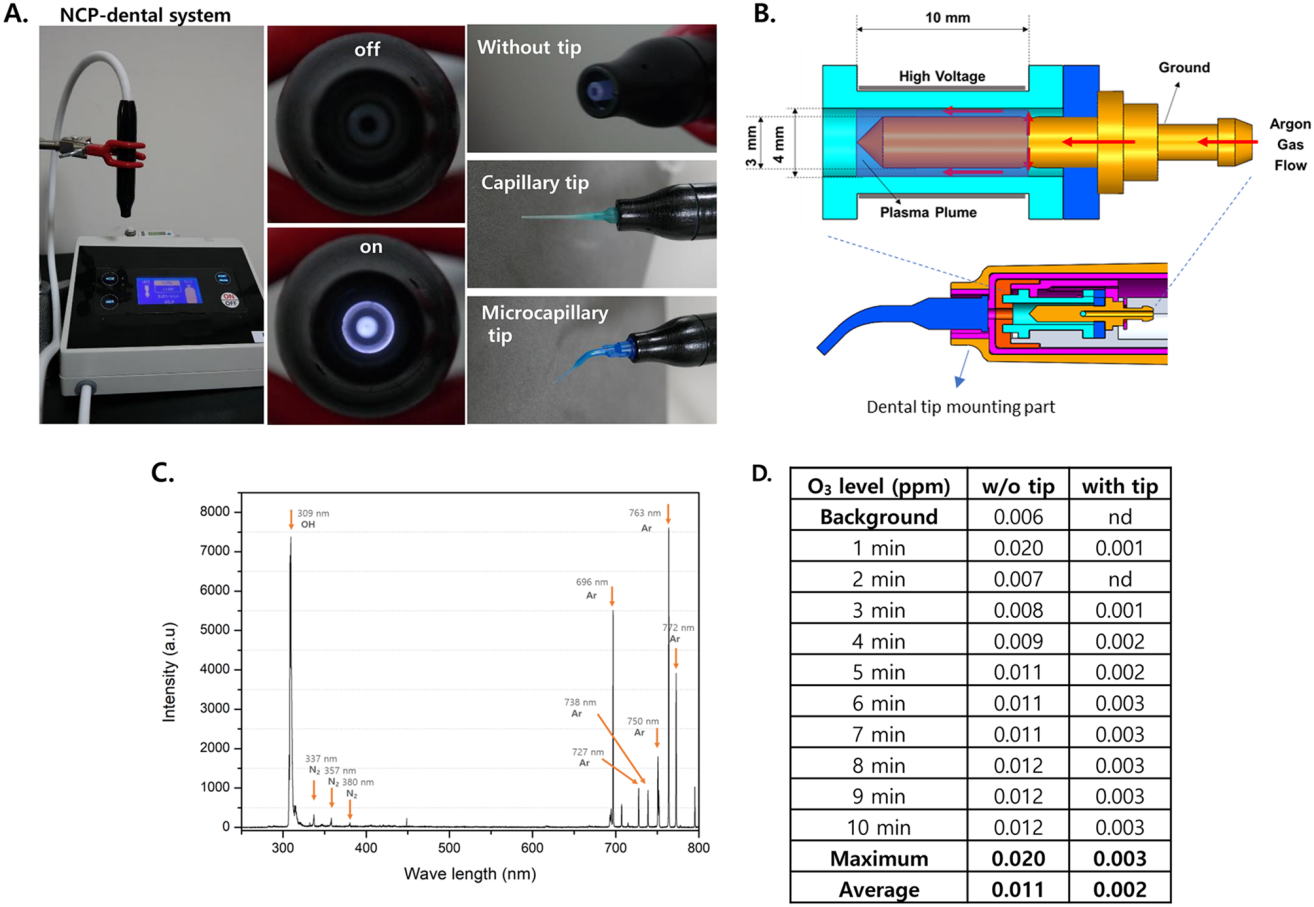
The NCP device can mount dental tips and produces argon plasma with low ozone. **(A)** The photographs of the NCP device developed and used in this study. This device can mount most of dental tips in the market. **(B)** A schematic diagram describing the structure of NCP generating part of the device. **(C)** The result of OES analysis of the plasma from the NCP device without dental tips. **(D)** The results of the measurement for the O_3_ level from NCP device. The measurements was performed for 10 min, and the O_3_ level was detected at every min. The distance between the gas inlet of O_3_ detector and the end of the NCP outlet (regardless of with or without capillary tip) was maintained at 1 cm.

To apply plasma to the oral cavity, which constitutes the respiratory system, a plasma-generating device that can minimise ozone generation must be developed. Hence, high-purity argon gas was used as a medium gas for plasma generation in the device developed in this study, and the device was designed to minimise the contact of oxygen with the plasma plume, as oxygen is essential for ozone generation. In addition, as shown in Fig. 1A, the device used in this study comprised one NCP generating module, which allows various dental tips to be mounted at the end of the NCP ejecting section.

To analyse the basic chemical constituents of the plasma formed in this device, an analysis using optical emission spectroscopy (OES) was adopted. As shown in Fig. 1C, a clear peak of OH radical with high intensity was monitored, and several clear peaks of argon ions with high intensity were monitored. On the other hand, only 3 peaks of excited N2 system with extremely low intensity was detected.

To analyse ozone generation, which an important objective in the development of this device, the ejected ozone level from the NCP device that changed based on the presence or absence of the dental tip was monitored. As shown in Fig. 1D, after measuring for 10 min without a dental tip attached, the maximum and average ozone concentrations were 0.020 and 0.011 ppm, respectively. However, the ozone level ejected from the NCP device mounted with dental tips was reduced to 0.003 ppm at maximum and 0.002 ppm on average.

### Surface treatment of NCP forms clear zone on four types of oral pathogenic microorganisms seeded on solid culture media in treatment-time dependent manner

**Figure 2:**
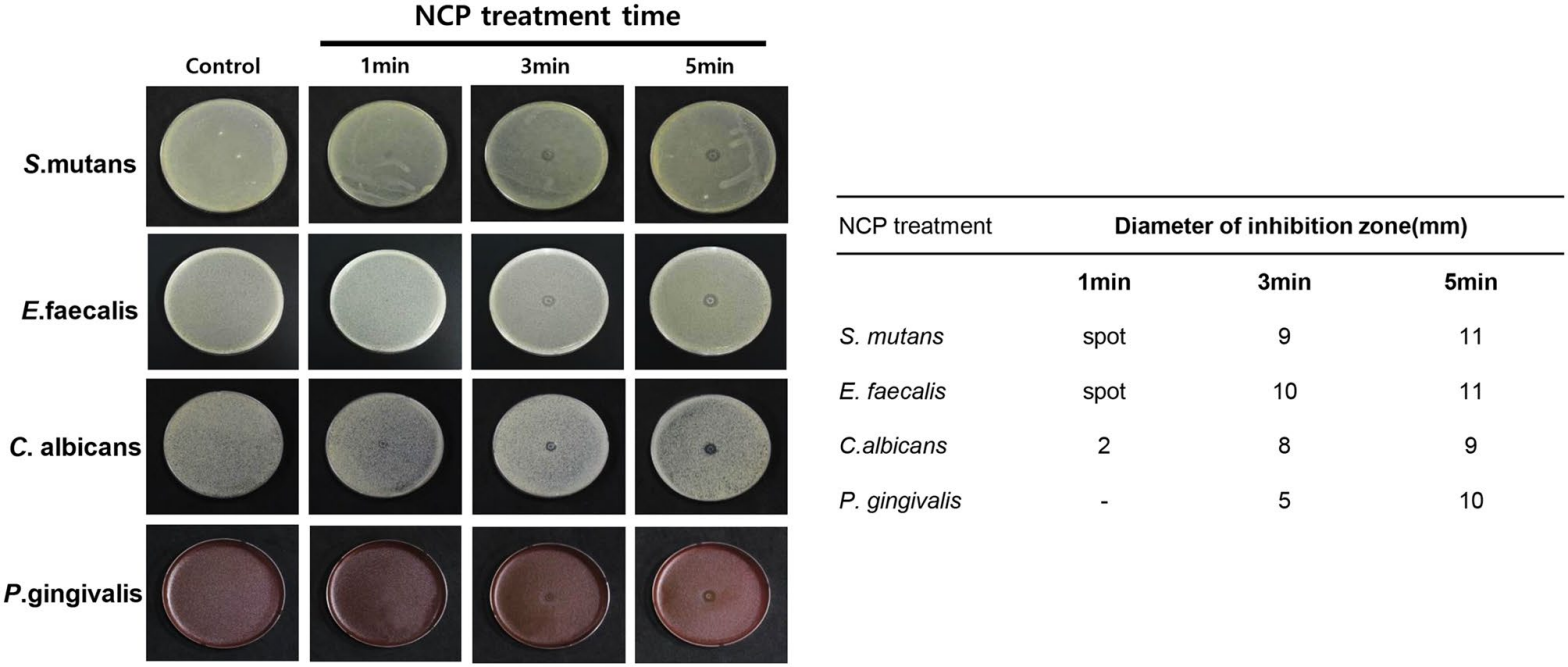
NCP treatment can kill 4 kinds of oral microbes on solid medium. The representative photographs showing the surface bactericidal effect of NCP against S. mutans, E.faecalis, C.albicans and P.gingivalis (left). The photographs were taken at 24 h after the NCP treatment for 1, 3 and 5 min. The distance between the end of NCP device and solid medium inoculated with oral microbes were maintained at 1 cm during the treatment. The diameter of inhibition zone were measured and presented in a table (right).

To investigate the immediate surface bactericidal efficacy of NCP against oral microbes, four types of oral microbes (*S. mutans, E. faecalis, C. albicans*, and *P. gingivalis*) smeared on the respective solid medium were treated using a NCP device (without dental tips) for 1, 3, and 5 min and then incubated for 24 h. As shown in Fig. 2, the treatment of *S. mutans* with NCP for 1 min resulted in a faintly inhibited growth area, whereas 3 and 5 min of NCP treatments resulted in clear zones measuring 9 and 11 mm in diameter, respectively. By contrast, the treatment of *E. faecalis* with NCP for 1 min resulted in a spot-like clear zone, and clear zones measuring 10 and 11 mm in diameter were formed via 3 and 5 min of NCP treatments, respectively. In the case of *C. albicans*, NCP treatment for 1 min was sufficient to create a 2 mm clear zone, and the diameters of the clear zones created via 3 and 5 min of NCP treatment were 8 and 9 mm, respectively. The *P. gingivalis* treated with NCP for 1 min did not indicate any growth inhibition, but 3 min of NTP resulted in a 5 mm clear zone, which increased to 10 mm after 5 min of NTP treatment.

### Effect of mounting dental tip on bactericidal effects of NCP against *C. albicans*

**Figure 3:**
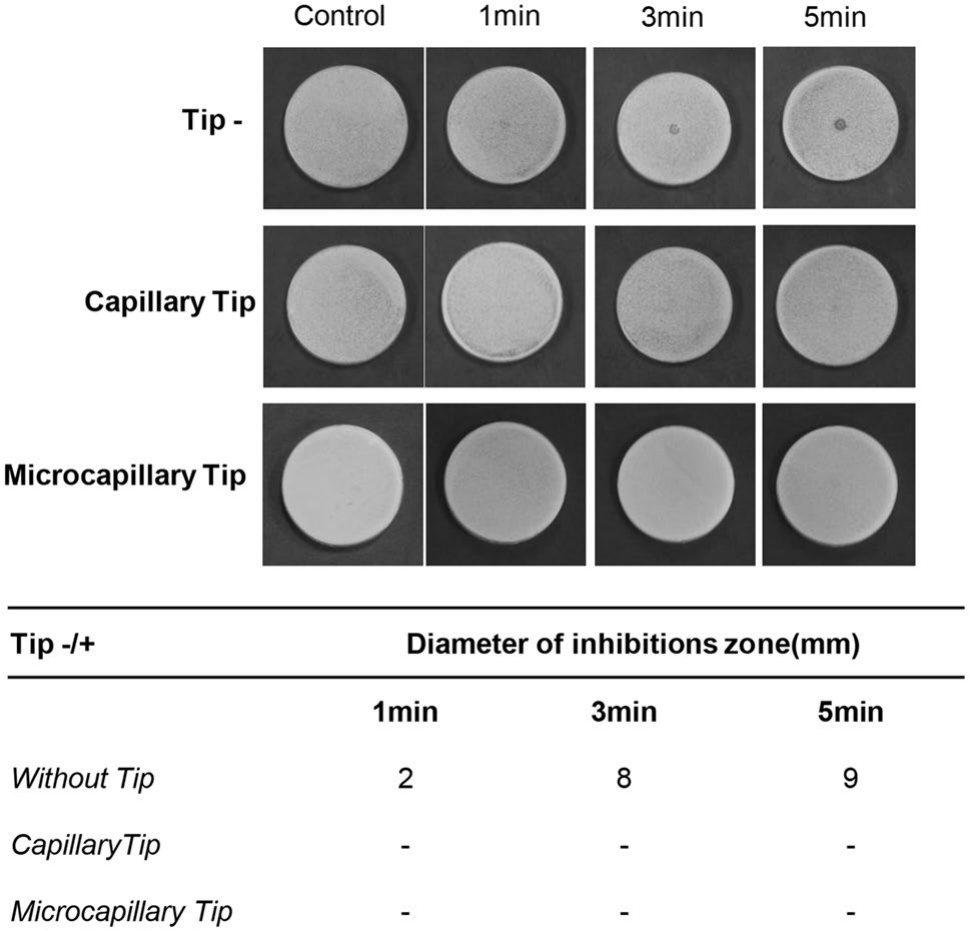
The mounting of dental tips reduced the bactericidal effect of NCP. The representative photographs showing the effect of dental tip (capillary and microcapillary tip) mounting on the bactericidal activity of NCP against C.albicans (upper panel). The photographs were taken at 24 h after the NCP treatment for 1, 3 and 5 min. The distance between the end of NCP outlet and solid medium were maintained at 1 cm during the treatment regardless of dental tip mounting. The diameter of inhibition zone were measured and presented in a table (bottom panel).

To apply the bactericidal effect of NCP to various types of dental procedures, dental tips that can access the fine gaps of the teeth must be applied. To determine whether the surface bactericidal effect of NCP was maintained when a dental tip was attached to the NCP device, the bacterial killing effect on *C. albicans* was analysed. As shown in Fig. 3, when the NCP was treated for 1, 3, and 5 min without a tip, clear zones measuring 2, 8, and 9 mm in diameters were formed, respectively. By contrast, when NCP treatment was performed after mounting two types of dental tips (capillary and microporous tips), no clear zone was formed even after the maximum treatment time of 5 min.

### Effect of NCP on growth of four types of oral microorganisms in liquid media

**Figure 4:**
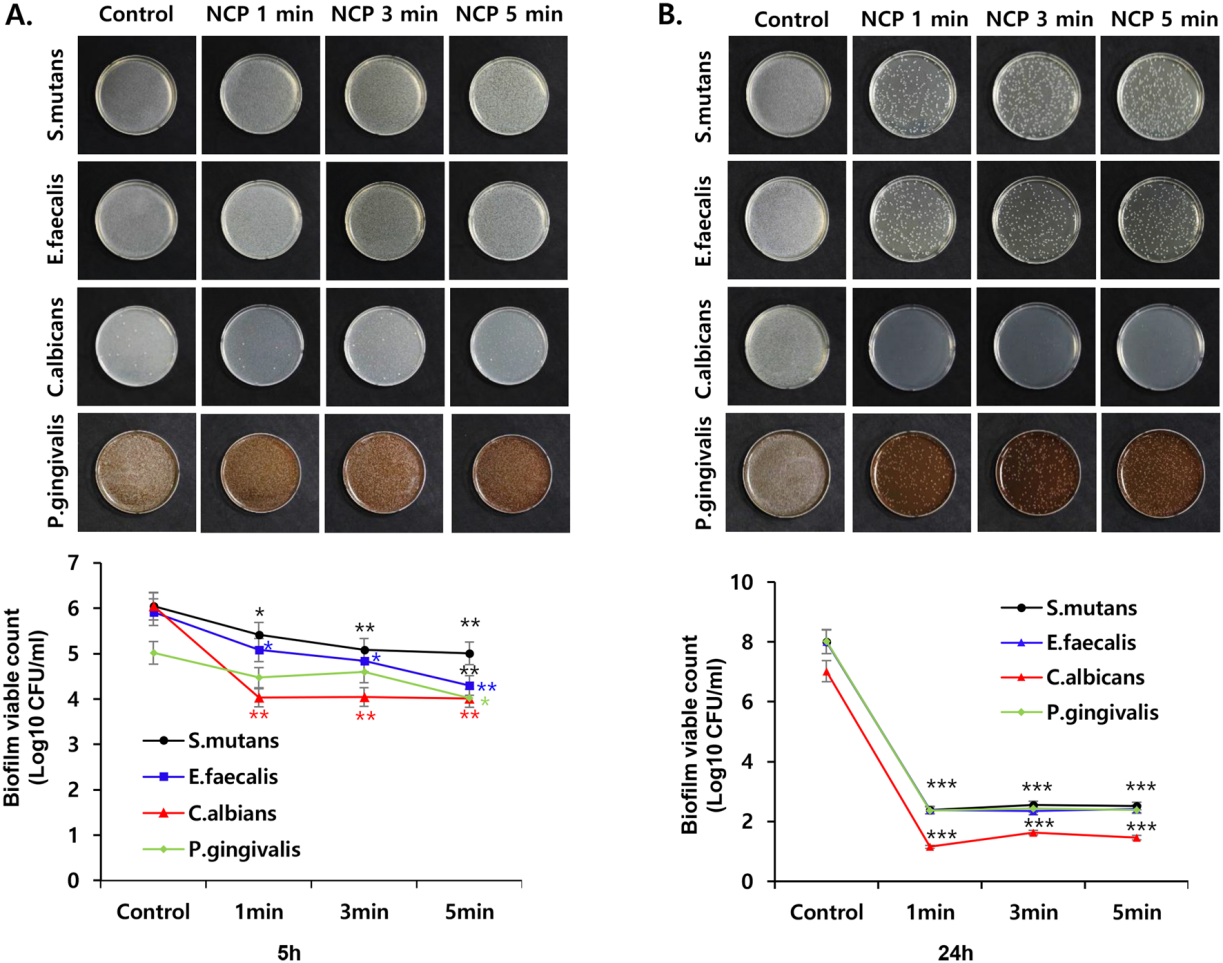
The bactericidal effect of NCP against 4 oral microbes in liquid medium. The results of experiments testing the sterilsation effect of NCP on *S. mutans, E.faecalis, C.albicans* and *P.gingivalis* in liquid medium. The medium containing oral microbes were subjected to 1, 3, and 5 min of NCP treatment respectively. After 5 h (**A**) and 24 h (**B**) of additional incubation in liquid medium, the samples were undergone a serial dilution, and plated on solid medium. At 24 h of further incubation, the representative photographs were taken (upper panel), and the number of colony was counted, and the results was presented as a graph (bottom panel). Data shown are the representatives of three independent experiments, *p < 0.05, **< 0.01, ***< 0.001.

To quantify the bactericidal efficacy of NCP against four types of oral microbes, the effect of NCP on the growth of oral microbes cultivated in liquid medium was analysed. The NCP was treated for 1, 3, and 5 min in 1 ml of the corresponding liquid medium that was inoculated with *S. mutans, E. faecalis, C. albicans,* and *P. gingivalis*, separately, and then incubated for 5 and 24 h. The cultured bacteria were diluted and inoculated into a solid medium, and colonies that formed 16 h later were counted to measure the colony forming units (CFU). As shown in Fig. 4A, the oral microbes that underwent 5 h of additional incubation after NCP treatment showed a moderate decrease in the CFU. Specifically, the CFU of *S. mutans* and *P. gingivalis* samples treated with NCP for 5 min decreased by only 1 log. The *E. faecalis* treated with NCP for 1, 3, and 5 min indicated approximately 1, 1.2, and 1.8 log of growth inhibition, respectively, whereas all *C. albicans* samples treated with NCP indicated approximate 2 log of growth inhibition. It was observed that the bactericidal effects of NCP on the four oral microbes became more prominent after an additional incubation for 24 h after the treatment. As shown in Fig. 4B, the NCP treatment for 1 min was sufficient for inducing approximately 5.5 log of growth inhibition in all four oral microbes. Furthermore, the additional treatment of NCP for more than 1 min did not promote the bactericidal effect of the NCP.

### Effect of mounting dental tip on bactericidal effect of NCP on oral microbes in liquid medium

**Figure 5:**
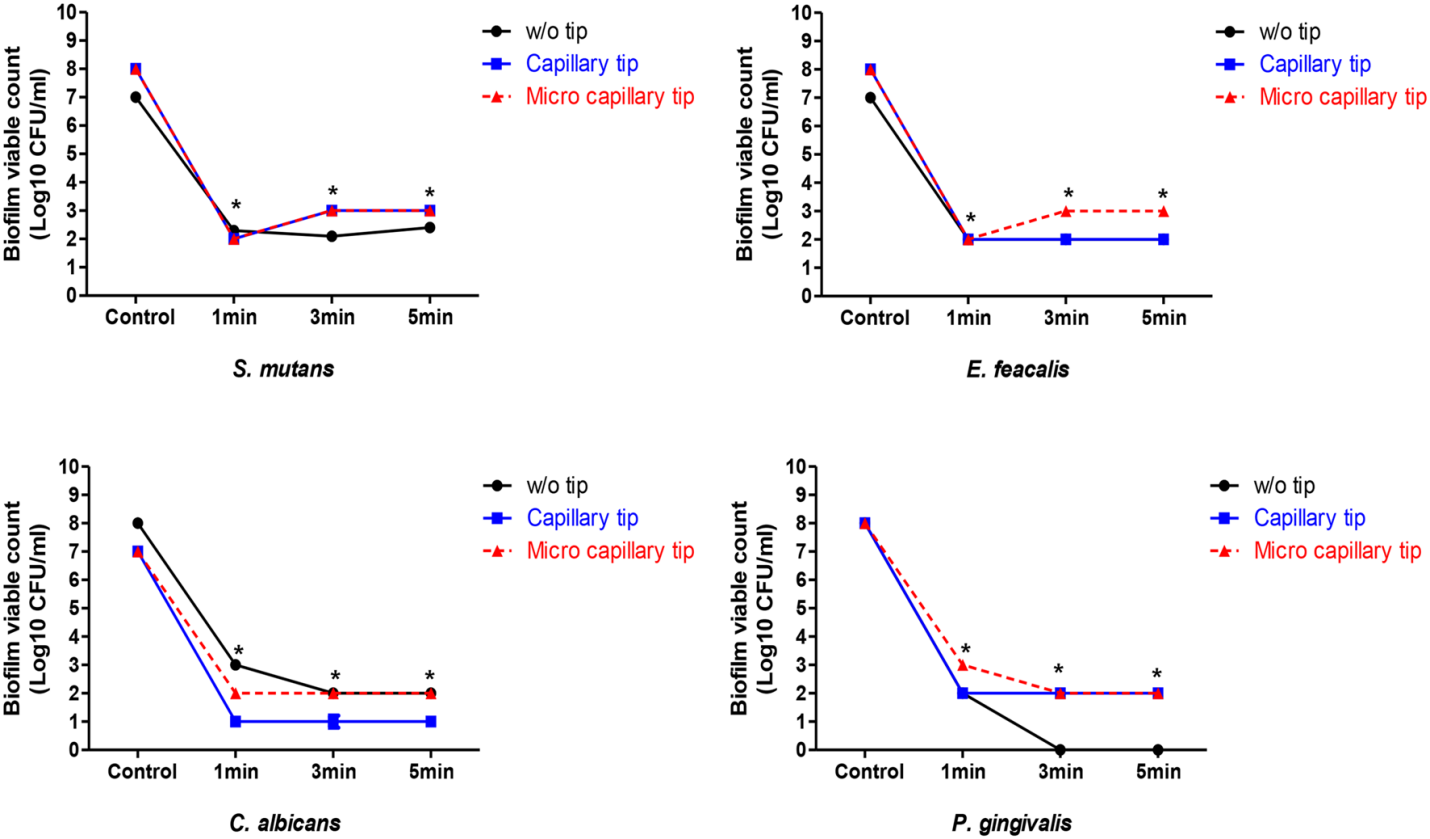
The effect of dental tip mounting on the bactericidal activity of NCP in liquid medium. Four kinds of oral microbes (*S.mutans, E.faecalis, C.albicans* and *P.gingivalis*) inoculated in the liquid medium were subjected to NCP treatment in the condition of unmounted or mounted with capillary or microcapillary tips. The treatment was performed for 1, 3 and 5 min, and the samples were incubated for 24 h further in the liquid medium. The samples were diluted serially, and plated on the solid medium. After 24 h, the colony forming units (CFU) were counted, and presented as a graph. Data shown are the average of three independent experiments, *p < 0.001.

Subsequently, we investigated the effect of mounting dental tips on the bactericidal effect of the NCP on the four oral microbes in liquid medium. As shown in Fig. 5, the NCP bactericidal effect against *S. mutans* was maintained even when the two types of dental tips were mounted on the NCP devices. In particular, treatment with mere NCP for 1, 3, and 5 min decreased the CFU of *S. mutants* by approximately 5.5 log, and this bactericidal effect was maintained when the device was mounted with capillary and microcapillary tips. In the case of *E. faecalis*, the CFU decreased by approximately 5 log after 1 min of NCP treatment without tips, but a 6 log CFU decrease was observed when the device was mounted with the two types of dental tips. In the case of *C. albicans* treated with mere NCP, the CFU decreased by approximately 6 log, but the bactericidal effect of the NCP decreased slightly to 5 log after the dental tips were mounted. The 5 min of dental tip mounted NCP treatment on *P. gingivalis* decreased CFU for about 6 log, but the mere treatment of NCP decreased the CFU of *P. gingivalis* for about 8 log.

### Role of reactive oxygen species in NCP-mediated growth inhibition of *S. mutans* in liquid medium

**Figure 6:**
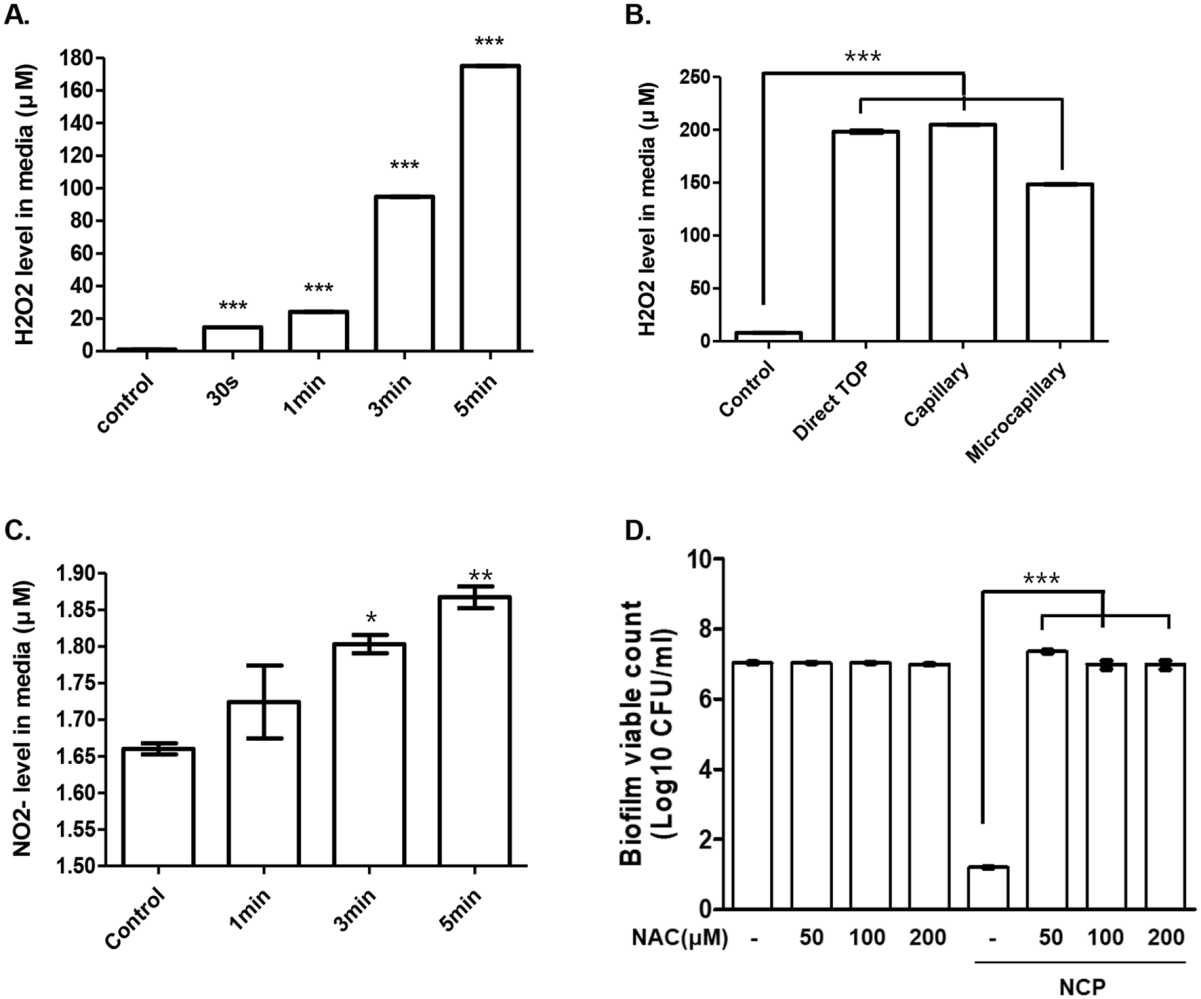
The NCP-mediated increase of H_2_O_2_ level in the medium is crucial for the bactericidal effect of NCP in liquid medium. (**A**) The results of the H_2_O_2_ concentration measurement. NCP was treated on 1 ml of liquid medium for 0.5, 1, 3, 5 min, and right after the treatment, the H_2_O_2_ level of the medium was detected using Amplex red assay kit. (**B**) The liquid medium (1 ml) samples were subjected to un-mounted NCP, NCP mounted with capillary or microcapillary tips for 5 min. After the treatment, H_2_O_2_ level in the medium was detected. (**C**) The results of nitrite level detection using Griess assay kit. The liquid medium (1 ml) samples were subjected to the NCP treatment for 1, 3, 5 min, and the NO^2−^ level in the medium was detected right after the treatment. (**D**) The results of the experiments monitoring the effect of NAC on the NCP-mediated bactericidal effect against S.mutans. The liquid medium samples containing *S. mutans* were treated with NCP for 5 min in presence or absence of NAC at concentration of 0.2, 0.4, and 1 mM respectively. The treated samples were incubated for 24 h further, and plated on solid medium. At 24 h after the additional incubation, CFU was counted. Data shown are the average of three independent experiments, **p < 0.05, **< 0.01, ***< 0.001.

To elucidate the mechanism of the NCP-induced bactericidal effect on oral pathogenic microbes in liquid medium, changes in active species generated in the liquid medium after NCP treatment were analysed. As shown in Fig. 6A, when the NCP was added to the microbial culture medium for 0.5, 1, 3, and 5 min, the H_2_O_2_ concentration of the medium changed to 15, 22, 95, and 175 μM, respectively. When monitoring the changes in the H_2_O_2_ concentration of the medium based on whether dental tips were mounted, a concentration of 205 μM was observed in the medium treated with capillary-tip-mounted NCP for 5 min, which was higher than that of the medium treated with unmounted NCP (180 μM). A relatively low concentration of 148 μM was observed in the NCP-treated medium equipped with a microcapillary tip (Fig. 6B).

Meanwhile, to analyse changes in reactive nitrogen species generated in the liquid medium during NCP treatment, the change in the concentration of nitrite (NO_2_^-^) in the liquid medium was analysed using the Griess assay. The result shows that when the NCP was treated in the liquid medium for 1, 3, and 5 min, the nitrite concentration increased gradually from 1.66 μM to 1.72, 1.8 and 1.87 μM, respectively (Fig. 6C).

To investigate whether the increase in H_2_O_2_ concentration in the liquid medium by the NCP is vital to NCP-mediated bactericidal under liquid conditions, an experiment using a reactive oxygen species (ROS) scavenger, i.e. N-acetylcysteine (NAC), was performed. As shown in Fig. 6D, the CFU of *S. mutans* samples treated with 50, 100, and 200 μM of NAC did not decrease, whereas treatment with NCP for 5 min inhibited the growth of *S. mutans* by approximately 6.8 log. Interestingly, when the NAC and NCP were treated simultaneously, the bactericidal effect of the NCP was fully recovered.

### Effect of dental tip length on surface bactericidal effect of NCP on solid media

**Figure 7:**
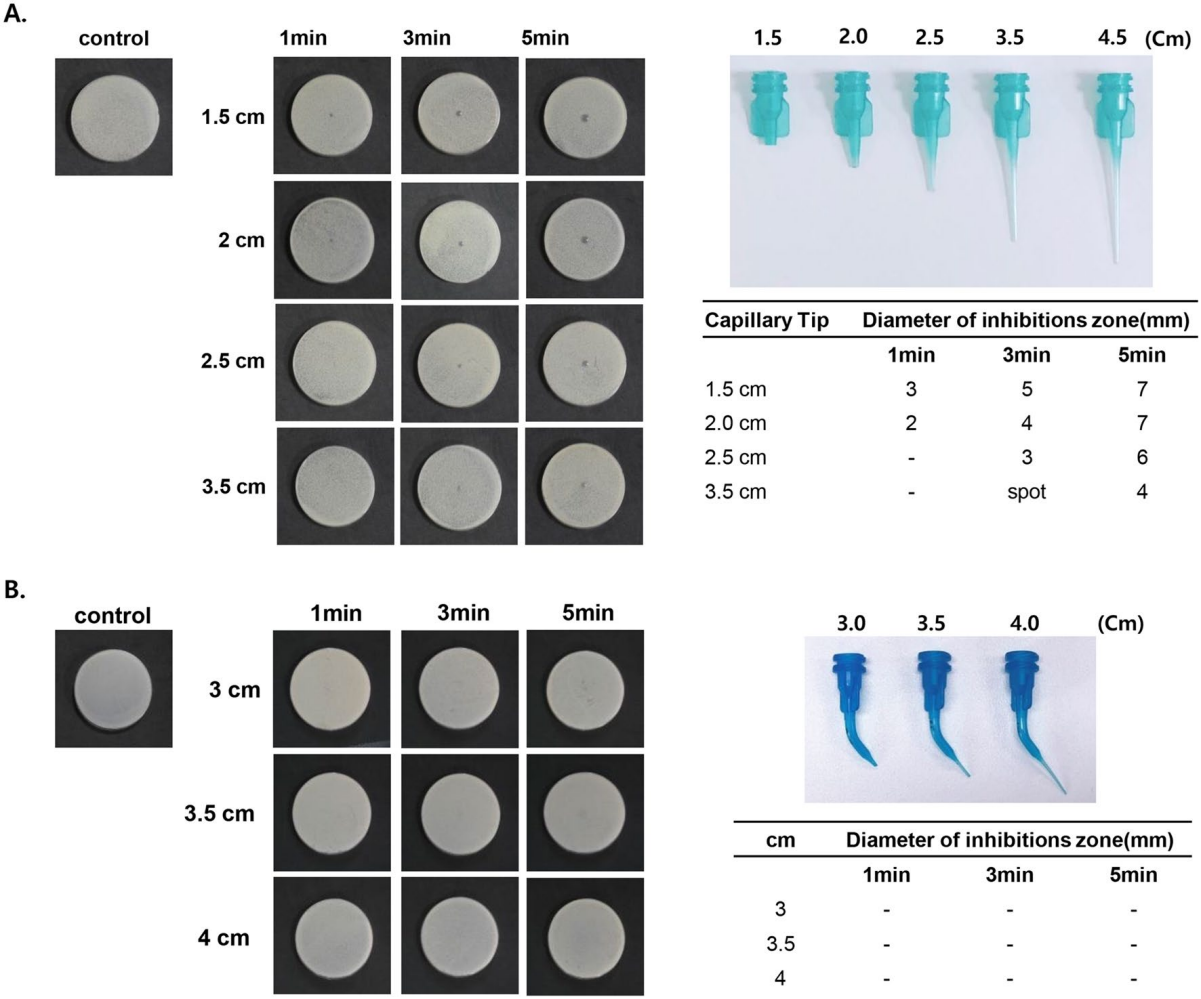
The effect of the dental tip length on the bactericidal effect of NCP mounted with dental tips. (**A**) The capillary tips (originally 4.5 cm in length) were cut into 1.5, 2, 2.5 and 3.5 cm, and mounted on NCP device. *S. mutans* inoculated on solid medium was treated with the NCP for 1, 3, 5 min. At 24 h after the treatment, the photographs showing a clear zone were taken, and the size of a clear zone was measured. (**B**) The microcapillary tips (originally 4 cm in length) were cut into 3 and 3.5 cm, and mounted on NCP device. The S.mutans on solid medium was subjected to NCP treatment for 1, 3 and 5 min. At 24 h after the treatment, the photographs showing a clear zone were taken, and the size of a clear zone was measured.

Earlier in this study, the fact that the surface bactericidal effect of NCP in a solid medium was not observed when the dental tip was installed indicates the possibility that the mechanism of the NCP in a solid medium might differ from that in a liquid medium. To elucidate the mechanism of NCP in solid medium, we analysed the possible effect of the dental-tip-mediated increase in the distance between the NCP generation section and the subject on the bactericidal effect of NCP. As shown in Fig. 7A, when the capillary tip was mounted, the existing 4.5 cm tip was cut into lengths measuring 1.5, 2.0, 2.5, and 3.5 cm; subsequently, each tip was mounted on the NCP device and then treated for 1, 3, and 5 min on *S. mutans* on the solid medium. Consequently, the 3.5 cm tip mounted on the NCP device and treated for 5 min yielded a clear zone measuring 4 mm, whereas a spot of clear zone was formed by 3 min of treatment; however, the 1 min treatment did not indicate any bactericidal effect. The size of the clear zone yielded by the treatment with a 2.5-cm-tip-mounted NCP device for 5 and 3 min were 6 and 3 mm, respectively; however, the 1 min treatment did not yield a clear zone. The 1 min treatment on the NCP device mounted with 1.5 and 2.0 mm tips yielded a clear zone measuring 3 and 2 mm, respectively. The clear zones yielded by 3 and 5 min of treatments with 1.5 cm tips measured 5 and 7 mm, respectively, whereas they were 4 and 7 mm when 2.0 cm tips were used, respectively.

However, the shortening of the microcapillary tip failed to recover the bactericidal effect of the NCP. As shown in Fig. 7B, the NCP treatment with microcapillary tips for three different lengths (4, 3.5, and 3.0 cm) did not yield a clear zone even after 5 min of treatment.

### Charged particles removable by grounded electric mesh are essential for NCP-mediated surface bactericidal effect

**Figure 8:**
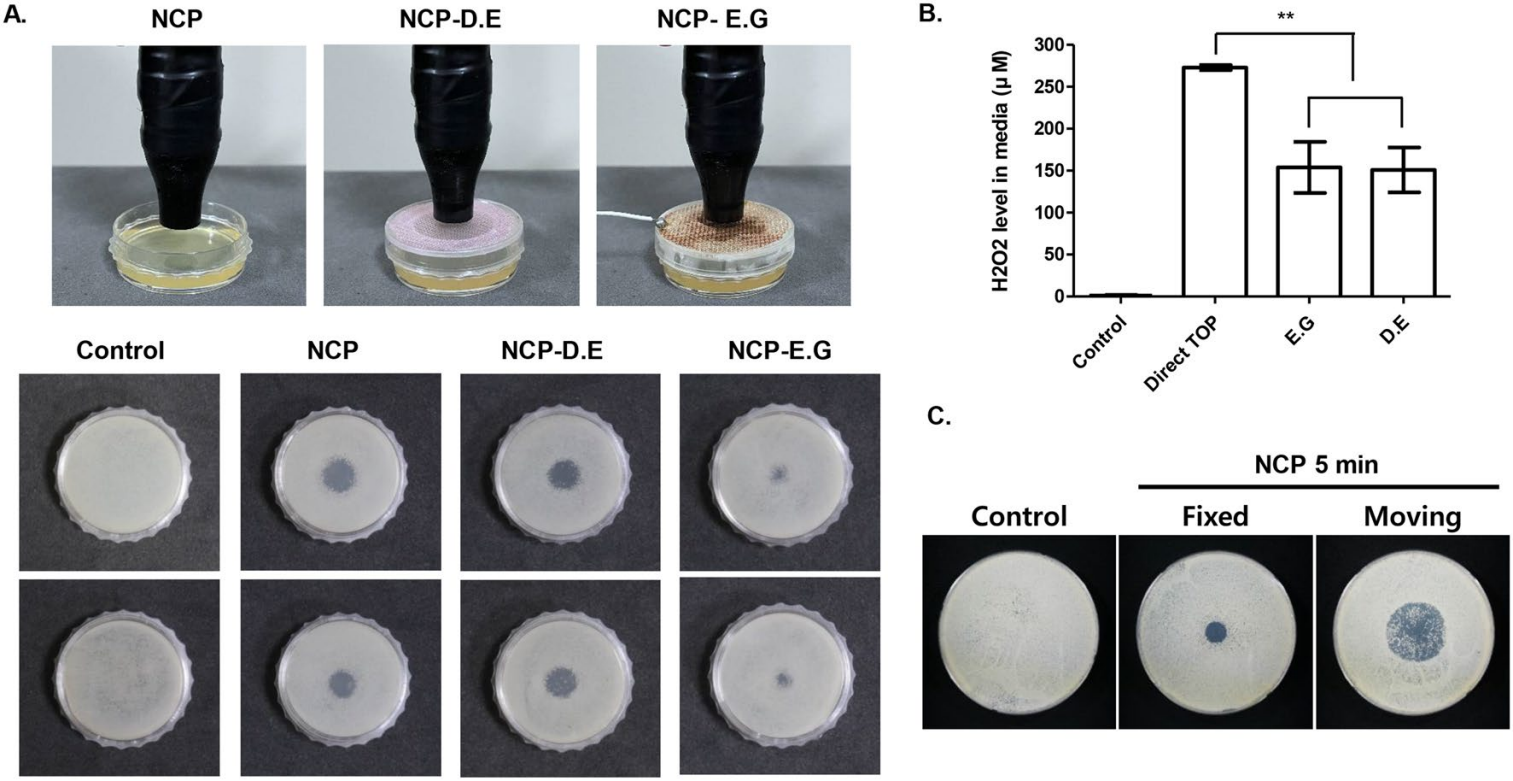
The charged particles of NCP is critical for the NCP-mediated bactericidal in solid medium. (**A**) *Upper panel* The photographs showing the 3 different NCP treatment conditions. NCP-D.E represents the NCP treatment condition placing a dielectric mesh between the target and NCP device, and NCP-E.G. means a grounded electric meshes were placed during the treatment. Bottom panel: The photographs showing the bactericidal effect of NCP in 3 different treatment conditions. The distance between NCP device and the S.mutans inoculated on solid medium in all three treatment conditions were maintained as 1 cm. At 24 h after the 5 min of NCP treatment, the photographs were taken. (**B**) The effect of 3 different NCP treatment methods on H_2_O_2_ level in the liquid medium. 1 ml of liquid medium was treated with NCP for 5 min using 3 different treatment methods described in (**A**). Data shown are the average of three independent experiments, **< 0.01. (**C**). *S.mutans* inoculated on solid medium was subjected to NCP using two treatment methods. Fixed treatment means NCP device was fixed at the middle of the plate during 5 min of treatment as usual, whereas moving treatment means the NCP was treated in moving manner during 5 min on the circle sized in 3 cm in diameter, keeping 1 cm distance. The photoghraphs showing a clear zone were taken at 24 h after the NCP treatment.

To determine the key element of NCP that contributes to the surface bactericidal effect, two types of meshes were installed between the NCP device and *S. mutans* inoculated on a solid medium. Subsequently, the size of the clear zone yielded after the NCP treatment for 5 min was monitored. As shown in Fig. 8A, the NCP treatment on *S. mutans* through dielectric mesh (NCP-D.E) demonstrated a similar microbial surface bactericidal effect to that of the NCP treatment without the mesh. However, when a grounded electric mesh was installed between the microorganism and the NCP device (NCP-G.E), the size of the clear zone generated after 5 min of NCP treatment reduced significantly.

To determine whether the change in the NCP surface bactericidal effect by the installed mesh type was due to the change in H_2_O_2_ generation, two types of meshes were installed when the NCP was treated for 5 min in the liquid medium, and the H_2_O_2_ concentration changes in the media were monitored. Based on the results shown in Fig. 8B, approximately 250 μM of H_2_O_2_ was detected in the medium treated with NCP without installing a mesh; however, only approximately 150 μM of H_2_O_2_ was detected in the NCP-treated medium when both types of meshes were installed. This result suggests that the surface bactericidal effect of the NCP is due to the charged particles (ions and electrons) that can be removed by the grounded electric mesh. Therefore, the possibility of broadening the effective range of NCP-mediated bactericidal by shifting rather than fixing at one point during 5 min of NCP treatment was investigated. As shown in Fig. 8C, a 5 min NCP treatment under the fixed condition yielded a clear zone measuring approximately 1 cm, whereas a larger but pale clear zone was formed when NCP treatment was performed in a moving manner on a circle measuring 3 cm in diameter.

### Surface bactericidal efficacy of NCP equipped with dental tip depends on diameter and length of tips

**Figure 9:**
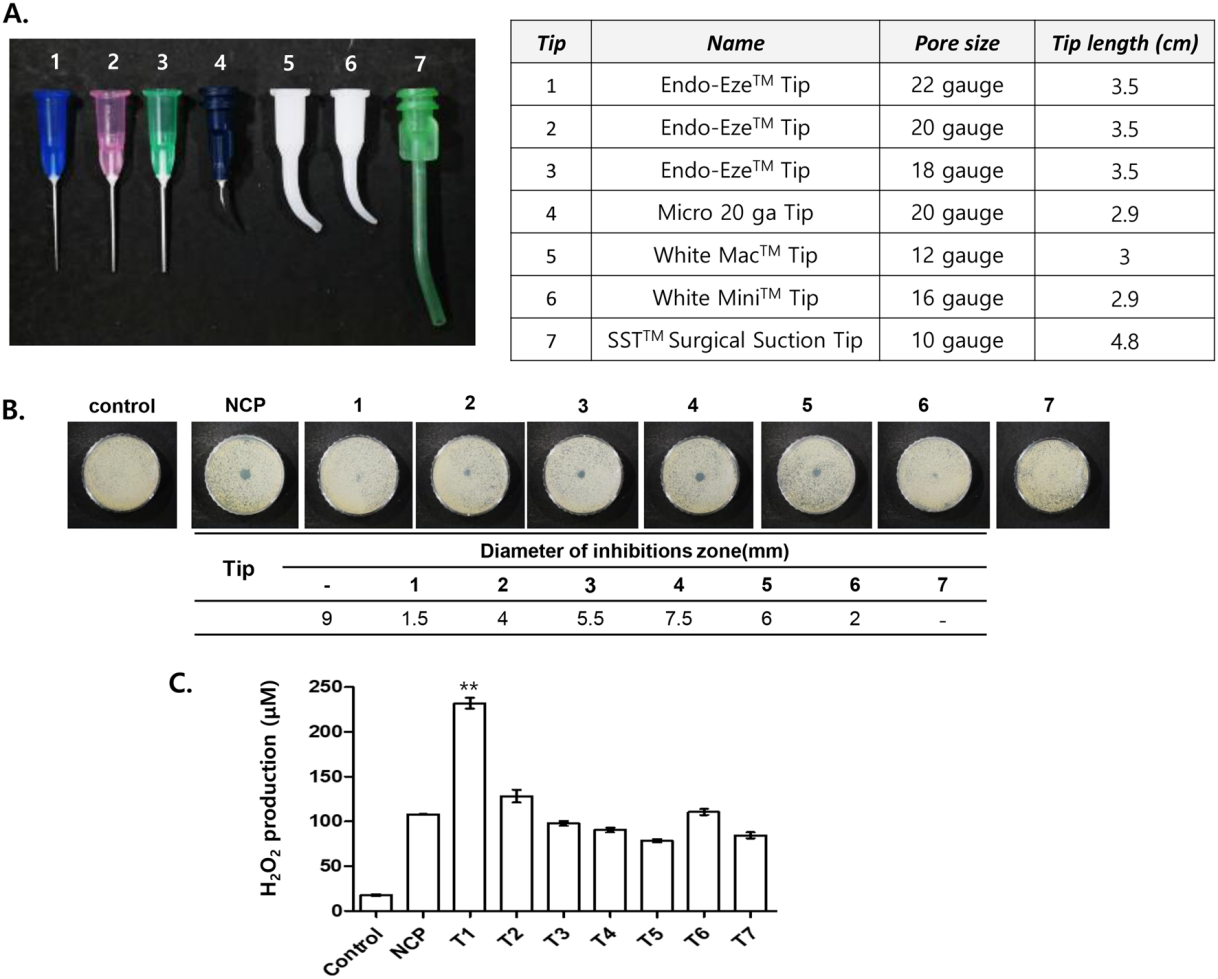
The bactericidal effect of NCP mounted with dental tips is dependent on the pore size and the length of tips. (**A**) Total 7 kinds of dental tips that can be mounted on NCP device were prepared. *Left panel* a photograph showing the appearance of 7 tips. *Right panel* a table showing specific information of the tips. (**B**) *S.mutans* samples on solid medium were subjected to NCP treatment for 5 min in unmounted or mounted with 7 kind of tips respectively. *Upper panel* A photographs showing a clear zone formed by 5 min of NCP treatment. *Bottom panel*: a graph showing the size of a clear zones. (**C**) The H_2_O_2_ level change of 2 ml of liquid medium after NCP treatment mounted with 7 kinds of dental tips. Data shown are the average of three independent experiments, **< 0.01.

To apply NCP to various dental procedures, the surface bactericidal effect must be maintained even when the NCP device is mounted with various types of dental tips. To secure the condition of the dental tip that allows the bactericidal effect of NCP to be maintained, various types of dental tips were mounted on the NCP device, and the bactericidal efficacy of NCP against *S. mutans* was investigated. Various types of dental tips that are actively used in the field of dentistry were prepared, and the pore size and length of the tips were analysed, as shown in Fig. 9A. Subsequently, each dental tip was mounted on the NCP device, and a 5 min NCP treatment was performed at fixed conditions, where a 1 cm distance was maintained between the tip end and the solid medium inoculated with *S. mutans*; a clear zone was formed, and its size was monitored 24 h after the treatment. As shown in Fig. 9B, although dental tips numbered 1 to 3 were of the same tip material and length, NCP treatment with tip number 1 (22 gauge) failed to form a clear zone, whereas that mounted with tip numbers 2 (20 gauge) and 3 (18 gauge) yielded clear zones, indicating the importance of the pore size. The NCP treatment with tip number 4, which was a 20-gauge-pore bent metallic tip, yielded a clear zone. In the NCP treatments with tip numbers 5 and 6, i.e. plastic tips of the same forms, the bactericidal effect of NCP was maintained when the device was mounted with tip number 5 (12 gauge), and a 6 mm clear zone was yielded; however, the size of the clear zone on the samples treated with tip number 6 (16 gauge) was 2 mm. Although tip number 7 had the largest pore size (10 gauge) among the tips tested, the NCP treatment using this tip did not yield a clear zone.

To confirm that the difference in the bactericidal effect of dental tips mounted on NCP is not dependent on H_2_O_2_, the liquid medium was subjected to treatment for 5 min under the same experimental conditions. As shown in Fig. 9C, the medium treated with NCP device mounted with tip number 1 showed the greatest increase in H_2_O_2_ concentration (approximately 230 μM); however, the NCP treatment involving tip number 1 on *S. mutans* did not yield a clear zone. Furthermore, the H_2_O_2_ concentration of each sample did not indicate any correlation with the bactericidal efficacy of the NCP device mounted with dental tips, as shown in Fig. 9B.

## Discussion

The impressive bactericidal effect of low-temperature plasma against various microorganisms was reported as a representative medical effect of the plasma^41^. This effect is being actively applied in plasma steriliser development for surgical tools and daily products^26^, as well as in the development of plasma food disinfection systems for more effective food storage^42^. Meanwhile, in terms of the development status of plasma sterilisers that can be directly applied to the human body, plasma wound healing devices that can promote healing by disinfecting the surface of skin wounds are being actively developed; however, the development of dental plasma devices that can directly treat oral cavities containing various pathogens are rare.

In this study, ozone, which can be harmful to the respiratory system despite its prominent bactericidal effect, was regarded as a major obstacle to the application of low-temperature plasma in the oral cavity because it can be produced during plasma generation. According to Gaens et al., when oxygen molecules in the air collide with high-energy electrons produced during plasma generation, oxygen molecules can be decomposed into atomic oxygen, and ozone can be formed when this atomic oxygen combines with nearby oxygen molecules^43^. Therefore, to minimise ozone generation, argon was used as a medium gas in the plasma-generating device used in this study, and this device was custom designed such that oxygen in the air cannot be accessed in the plasma generation section. In addition, to deliver the numerous biological properties of plasma into the complex structured target within the oral cavity, this device allows various types of dental tips to be mounted. As shown in Fig. 1, this device effectively generates low-temperature plasma, and the results of OES analysis in this study confirmed that this plasma included OH radicals and excited argon through the unique argon plasma chemical reaction. Since we used high purity argon gas, the peak of OH radical in this OES analysis results might come from H_2_O, which might be smeared into the NCP device gas inlet during the device was not under operation. According to our Supplementary Data 1, which were performed in winter season, the OH peak at initial time point of NCP generation was much smaller than that of Fig. 1C, the results of OES performed in humid rainy summer season. In addition, looking at the results of OES analysis of NCP measured at 2, 5, 10, and 20 min of plasma generation, the OH peak decreased as the measurement time elapsed. This result strongly suggests the fact that although our NCP system has unique structure which cannot permit an entry of ambient air during operation, ambient air containing H_2_O can enter into gas path of the device in stop mode, and be a cause of OH radical in the system. In order to confirm whether the plasma generation technology developed in this study can suppress ozone generation, an experiment using an ultra-precision ozone meter was conducted. The result indicates that when plasma was generated after using the device for 10 min, the maximum ozone generation concentrations at a distance of 1 cm from the gas outlet measured 0.02 and 0.0003 ppm in the absence and presence of a dental tip, respectively. The allowable atmospheric ozone level indicated in the Air Quality Guidelines prepared by the World Health Organization is less than 0.051 ppm (100 μg/m^3^); therefore, the ozone level generated from this device was more than two times lower than that stipulated by the guideline in the unmounted condition, and more than 10 times lower in the dental tip mounted condition. These results indicate that this noble plasma-generating technology poses minimal risks from ozone. The unique structure of NCP device also limited the generation of NO and NO_2_ those can be toxic to human tissue. As our Supplementary Data 2 shows, NO from NCP device was not detected, and the maximum level of NO_2_ was about 0.007 ppm during 10 min of NCP generation period. In addition, the temperature of the plasma gas ejected from this device was measured at 27.0 °C and 25.7 °C when the dental tip was not mounted and mounted, respectively. Because this plasma-generating technology generates plasma that do not induce thermal damage to oral tissues while maintaining an extremely suppressed ozone level, this technology was named NCP technology.

The bactericidal effect of low-temperature plasma against various oral pathogens has been reported by various researchers. However, various plasma generation methods exist, and most studies have used plasma devices that generate plasma with much higher plasma temperatures and ozone levels than NCP^38–40,44,45^. Therefore, in this study, four types of representative oral pathogenic microbes (*S. mutans*, *E. faecalis, P. gingivalis*, and *C. albicans*) were used to investigate the bactericidal effect of NCP. As shown in Fig. 2, the NCP effectively killed all four oral microbes on solid medium. It is noteworthy that (1) the oral microbes were not directly in contact with the plasma glow; instead, they were only exposed to the plasma afterglow of the NCP. (2) Additionally, although the ozone level and gas temperature of the NCP remained at the toxic level, the NCP effectively killed the oral microbes. We conducted a study to confirm whether the surface bactericidal effect of NCP can be maintained even when the dental tip is mounted. The dental tips selected were capillary and microcapillary tips, which are the most frequently used tips to suction or irradiate the byproducts of pockets created during various dental procedures. It was observed that the surface bactericidal effect of the NCP on *S. mutans* diminished when the dental tips were mounted (Fig. 3); in fact, this phenomenon was also observed in the other three oral microbes (data not shown).

To quantify the bactericidal efficacy of NCP against the four types of oral microbes, the effect of NCP on the growth of oral microbes in a liquid medium was analysed. Five h after the NCP treatment, the CFU of *S. mutans*, *P. gingivalis*, and *E. faecalis* decreased by approximately 90% only when the microbes were exposed to 5 min of NCP, whereas 1 min of NCP treatment was sufficient to achieve an approximately 100 fold decrease in the CFU of *C. albicans*. These results indicate that *C. albicans* is the most sensitive to NCP among the four oral microbes investigated. It was observed that 24 h after the NCP treatment, a 1 min NCP treatment was sufficient to decrease the CFU of the four types of oral microbes by more than 5 log. In contrast to the results of the NCP surface bactericidal effect on solid medium, the bactericidal effect of NCP against oral pathogens in liquid medium was maintained even when the device was mounted with capillary and microcapillary tips (Fig. 5). These findings suggest that the surface bactericidal action of NCP against oral pathogenic microbes in solid medium and its bactericidal action in liquid medium can be achieved via different mechanisms.

The results of several experiments confirmed that NCP-mediated bactericidal effects in liquid medium was primarily due to increased H_2_O_2_ levels in the medium after the treatment. As shown in Fig. 6, the treatment of NCP on liquid medium increased the H_2_O_2_ concentration as a function of the treatment time. Furthermore, the H_2_O_2_ concentration in the medium treated for 5 min with an NCP device mounted with a capillary tip was approximately 200 μM, which was similar to the result for the medium treated with an NCP device without tips. However, the NCP-mediated increase in H_2_O_2_ concentration decreased slightly when the microcapillary tip was mounted (the H_2_O_2_ concentration of the medium was approximately 150 μM). However, because a 1 min NCP treatment was sufficient for a 5 log decrease in the CFU, the minimum H_2_O_2_ concentration for inducing a 5 log decrease in the CFU might be 20 μM. Therefore, this microcapillary-mediated decrease in H_2_O_2_ concentration did not affect the bactericidal effect of NCP in liquid medium. In addition, the results of a study using NAC, an effective scavenger of ROS, indicated that the bactericidal effect of NCP on *S. mutans* was completely diminished in liquid medium containing NAC. In fact, these results indicate that the bactericidal effect of NCP in liquid medium was due to the increase in H_2_O_2_ concentration, which might induce oxidative stress by producing ROS. Finally, as our Supplementary Data 3 shows, the mere addition of H_2_O_2_ in the media at the similar concentration of the media treated with NCP for 1, 3, and 5 min showed similar antibacterial effects. Therefore, the key player of NCP’s antibacterial effect in liquid media must be increased H_2_O_2_. Although there are many report presenting elevated H_2_O_2_ level in the plasma treated liquid, but there are no clear answer for how and where H_2_O_2_ was generated by the plasma. This might be caused by the complexity of plasma chemistry those can promotes the increase of H_2_O_2_ in the liquid, and it can be diverse dependent on the plasma source, surrounding air condition, and the liquids^46^. In order to answer for where and how H_2_O_2_ in NCP-treated liquid medium was generated, several additional experiments those can measure several species in each phases (gas, liquid, solid phases). Unfortunately, we failed for to get exact answer to this question, but only can suggest one possible mechanism based on data of this study, and the reports of others. According to Soe et al., when non-thermal plasma using argon gas is generated, the surrounding H_2_O can be decomposed and OH radicals can be produced^45,47^. Various researchers also suggested that the OH radicals generated by plasma react with each other to produce H_2_O_2_ at the surface of a liquid^48,49^. In this study, because the NCP can generates gas phase OH radicals as shown in Fig. 1C, the NCP-mediated increase in H_2_O_2_ concentration in the medium might be regarded as a consequence of the action of OH radicals. Since the growth media used in this study contains several kinds of organic chemicals those can scavenge the OH radical, this conversion of OH from NCP device into H_2_O_2_ in liquid media is very important for NCP’s antibacterial effect in the liquid media. Moreover, although the NCP slightly increased the nitrite concentration in the liquid medium, it was believed that this small change in nitrite concentration did not significantly affect the killing efficacy of NCP.

In contrast to NCP-mediated bactericidal in liquid medium, our data confirmed that the surface bactericidal effect of NCP on solid medium was H_2_O_2_ independent. Based on the first experiment to elucidate the mechanism of the surface bactericidal effect of NCP in solid medium, we confirmed that shortening the capillary tips increased the size of the clear zone (Fig. 7). This indicates that the key element of NCP for surface bactericidal in solid medium can be weakened when the distance between the NCP device and target is increased. By contrast, when the microcapillary tip was used, the NCP treatment with a tip shortened by 3 cm did not yield a clear zone; this implies that in addition to distance, other factors can affect the bactericidal effect of the NCP on solid medium. In regard to the results from the experiments using two types of mesh (Fig. 8), it was discovered that in contrast to the dielectric mesh, which did not affect the bactericidal effect of the NCP, placing an electrically grounded mesh between the NCP device and *S. mutans* inoculated on the solid medium reduced the NCP’s bactericidal activity. Because this electrically grounded mesh can hinder the action of charged particles of NCP, such as electrons and ions, gas phase OH radicals can pass through this mesh. This indicates that the surface bactericidal effect of NCP on solid medium is mediated by the charged particles of NCP, not gas phase OH radicals from NCP device. Furthermore, the H_2_O_2_ concentrations of the liquid medium treated with NCP in presence of dielectric mesh and electric grounded mesh were slightly decreased from that of non-mesh condition, but they were similar to each other. Likewise, in our Supplementary Data 6A, NCP treatment on solid medium increased H_2_O_2_ level at the surface of the medium, and this effect was not modulated by two kinds of meshes. In addition, the mere addition of a H_2_O_2_ drop at concentration of 20, 50, 100 and 200 μM on *S. mutans* cultured on the solid medium did not showed any antibacterial activity (Supplementary Data 6B). Hence, these results imply that the bactericidal effect of NCP on solid medium is H_2_O_2_ independent. Furthermore, the possibility that the limited size of the NCP-mediated clear zone is due to the short working time of the charged particles was investigated. As shown in Fig. 8C, when *S. mutans* was treated with NCP for 5 min in the moving mode, the size of the clear zone increased compared with that of the sample treated with NCP in the fixed mode. This indicates that the charged particles from the NCP propagated along with the gas flow and killed the oral microbes only in the area where they collided directly, and that the bactericidal ability weakened during spreading after the collision.

Finally, to identify a method to maintain the surface bactericidal effect of NCP on solid medium in dental tip-mounted conditions, various types of dental tips (i.e. of different materials, shapes, thicknesses, and lengths) were mounted on an NCP device, and the bactericidal effects were investigated (Fig. 9). The result shows different bactericidal efficacies of NCPs on *S. mutans* depending on the type of dental tip mounted on the NCP device. In particular, the treatment involving an NCP device mounted with shorter dental tips with a low gauge value (larger pore size) indicated more prominent surface bactericidal effects. In our previous results (Fig. 3), the surface bactericidal effect of the NCP diminished when capillary and microcapillary tips were mounted; this might have been caused by the extremely small pore sizes (27 gauge for capillary tip; 32 gauge for microcapillary tip) and the extremely long tips. In general, at the beginning of the argon plasma generation process, electrons escaped from argon, thereby generating argon ions and electrons. According to Dobrynin et al. and Fridman et al., the charged particles of plasma are crucial in non-thermal plasma-mediated bactericidal as they can rupture the outer membrane of bacterial cells^50,51^. Mendis et al. argued that when microorganisms were treated with plasma, the charged particles of plasma can accumulate on the surface of the membrane, thereby resulting in a ruptured membrane^52^. Meanwhile, according to Digel et al., the charged particles of plasma form OH radicals on the surface of the microbial membrane, thereby inducing chemical modification on the surface protein and hence microorganism death^53^. In this study, the surface bactericidal effect of NCP that was weakened by the electrically grounded mesh suggests that the surface bactericidal effect of NCP was due to argon ions or the electrons of NCP. The results of Fig. 9 can be regarded as caused by the decreased amount of charged particles due to the bottleneck phenomenon at the smaller sized pore of dental tips, which can result in collisions between argon ions and electrons. Even though the pore size of the tip is sufficiently large, if the length of the tip is extremely long, then the energy state of argon ions and electrons will decrease, and the probability of collision will increase, thereby weakening the bactericidal activity of NCP. The fact that the H_2_O_2_ concentration of the liquid medium after the NCP treatment did not coincide with the surface bactericidal efficacy of the NCP device mounted with each dental tip also suggests the fact that the surface bactericidal effect of NCP was not due to the NCP-mediated increase of H_2_O_2_. The fact that the decrease of air humidity, which can cause the decrease of H_2_O_2_ level in NCP-treated media (Supplementary Data 4), did not hindered antibacterial activity of NCP in solid medium (Supplementary Data 5) also support the fact that NCP’s bactericidal effect on solid medium is H_2_O_2_ independent.

In this study, a new plasma-generating device, i.e. the NCP device, which produces a cold plasma that does not cause thermal damage to oral tissues keeping extremely low ozone production rate, was developed to apply various medical effects of plasma. This study not only confirmed that the NCP from this device effectively killed four major oral pathogenic microorganisms, but also elucidated that the bactericidal effect of NCP was H_2_O_2_ (which were produced by OH radical of the NCP) dependent in liquid medium, and that charged particles of NCP were vital to the surface bactericidal effect on solid medium. In addition, we confirmed that the bactericidal effect of NCP can be maintained even when the device was mounted with certain types of dental tips. Therefore, based on this study, NCP technology is expected to be applicable to various dental procedures in the near future.

## Methods

### Plasma generating device

Figure 1A shows the NCP-dental system, which were developed by FEAGLE corporations. This plasma device has a main body, which was composed with smps, solenoid valve, gas flow rate controller, high voltage circuit, etc., and it has a handpiece, the part generating the NCP. Inside the handpiece (Fig. 1B), there are a coaxial DBD (Dielectric Barrier Discharge) type plasma source, which consists of a stainless steel (stainless steel 306) inner electrode and an outer electrode (Al_2_O3) surrounding the outer diameter of the ceramic nozzle. The end of the plasma generating nozzle of the handpiece was designed to mount various type of dental tips by configurating a luer lock type fastening part (Fig. 1B). When the start button of the main body is pressed, argon gas flows between the inner electrode and the ceramic nozzle, and an output voltage of 3 kVpp with a frequency of 20 kHz is applied to the electrodes to generate plasma. The argon gas rate of this device can be regulated through gas flow rate controller, and in this study, the gas flow rate was fixed as 1 slm (standard litter per min).

### Optical emission spectroscopy (OES)

The emission spectra were measured using OES (optical emission spectrometer) equipment (Ocean Optics, USB2000+, USA) to analyze the reactive species generated inside the device during the plasma generation process. The light acquisition times were set as 200 ms, and the optical probe was placed at 10 mm distance from the end of the nozzle of plasma generator.

### Ozone concentration measurement

In order to measure the ozone concentration in the plasma gas ejected from NCP device, a Serinus 10 Ozone Analyzer (OA; Acoem Ecotech, Knoxfield, Victoria, Australia) was used. The OA aspirator was placed at approximately 10 mm in front of the plasma gas ejecting hole (end of handpiece or mounted), and the measurement was performed at every 1 min, for 10 min. The OA uses UV absorption to measure the concentration of ozone over time with an accuracy of 0.001 ppb within a 0–20 ppm range.

### Oral pathogenic microorganism culture

In order to test the antimicrobial effect of NCP on oral microbes, a total of 4 bacteria were used: *Streptococcus mutans* (KCTC 3065), Enterococcus faecalis (KCTC 3206), Candida albicans (KCTC 7678), and Porphyromonas gingivalis (KCTC 5352). All strains used in this study were distributed from the Korean Collection for Type Cultures (KCTC, Daejeon, Korea). As the specific culture medium for Streptococcus mutans and Enterococcus faecalis contains Brain heart infusion Broth (BD DIFCO, USA) was used, and Candida albicans was cultured in Yeast Mold Broth(BD DIFCO, USA) containing medium. These 3 microbes were cultured at normal 37 °C incubator. For Porphyromonas gingivalis, the Trypticase soy broth(BD DIFCO, USA) containing medium was used, and cultured at 37 °C chamber with anaerobic condition (5% CO_2_, 4.98% H2, 90% N_2_). After increasing vitality through the sub-culture (diluting 1 ml of bacteria in 4 ml of culture medium), all each microbes were cultured overnight at the 37 °C incubator, and before using the microbes for the experiments, the optical density of the bacterial suspension was set as 1 at 600 nm.

### Materials and reagents

Dental tips were purchased from Ultradent Product. 2 kinds of meshes (dielectric and electric grounded meshes) were designed and produced by Feagle Corporations, those can be placed at the top of the 35 mm cell culture dish. The dielectric mesh was produced using a cotton mesh, so that most of all working elements of NCP can be penetrate through it, although the flow rate of NCP can be reduced. On the other hand, the electric grounded mesh was made using a cupper mesh which was directly linked to the ground panel of power supply device, so that the effects of charged particles from NCP can be eliminated by this type of mesh. All chemicals were purchased from Sigma-Aldrich Korea unless otherwise indicated.

### Zone of growth inhibition (clear zone) measurements

In order to validate the surface bactricidal effect of NCP on 4 kinds of oral pathogens on the solid medium, all microbes were inoculated on each specific medium at concentrations of 1 × 10^8^ CFU/ml. Right after the inoculation, each culture dishes were subjected to the NCP treatment for 1, 3, 5 min. After 16 h of additional incubation, the photographs of a clear zone were taken, and the diameter of a clear zone was measured.

### Colony forming unit (CFU) measurements

To quantify the bactricidal effect of NCP on 4 kinds of oral pathogenic microbe, the changes of CFU after the NCP treatment was monitored. The microbes were inoculated in 1 ml of liquid growth medium and plated at 24 well cell culture dish. Then, the microbes were treated with NCP and dental tips mounted NCP for 1, 3, and 5 min, and incubated for 5 or 24 h further. When the microbes of non-treated control concentrations became 10^7^ CFU/ml, all the samples were serially diluted, and inoculated on the solid medium. After 16 h of additional incubation at 37 °C incubator, the photograph of culture dishes were taken, and the number of colony were counted.

### Amplex red assay

For monitoring the NCP-mediated changes of the H_2_O_2_ level in the liquid medium, the assay using the Amplex Red hydrogen peroxide/peroxidase assay kit (Invitrogen, Carlsbad, CA, USA) was adopted. The 1 ml of liquid medium was subjected to the NCP treatment for 0.5, 1, 3, 5 min, and for testing the effect of the mounting with Capillary tip and Micro capillary tips on NCP-mediated changes in H_2_O_2_ level, NCP treating time was fixed in 5 min. All the treated and non-treated samples were subjected the H_2_O_2_ assay as manufacturer’s instruction. Briefly, the 100 μl of samples were mixed with 100 μM Amplex Red reagent and 0.2U/mL HRP at 96-well plate. The samples were incubated for 30 min at room temperature in dark condition, and then OD values were measured using a Microplate reader (MultiSkan FC, Thermo Fisher Scientific K.K.) at 560 nm.

### Griess assay

To evaluate the effect of PC on the NO density within the cell growth medium, the Griess assay was adopted using a Griess Reagent Kit (Invitrogen, Eugene, OR, USA) according to the manufacturer’s protocol. In brief, 1 ml of liquid medium was added to a 35 mm cell culture dish and treated with NCP for 1, 3, and 5 min. After the treatments, 150 μl of non-treated and NCP treated medium was used for the Griess assay (in triplicate). Afer 30 min of incubation, the optical density of the medium was read by 96-well microplate reader at 548 nm.

### Data analysis

All numerical values of the experimental results were statistically processed using the SPSS program. Data are presented as ± standard error of the mean (SEM) of at least three independent experiments. The two-tailed Student’s t-test was used to assess statistical significance for differences in mean values, and the significance was set at p < 0.05.

## Supplementary Information

Supplementary Information.

## Data Availability

All data generated or analysed during this study are included in this published article.
