# Supplementary material for: No-ozone cold plasma can kill oral pathogenic microbes in H2O2-dependent and independent manner
Source: Sci Rep. 2022 May 9;12:7597. doi: 10.1038/s41598-022-11665-z (PMC9085805; doi:10.1038/s41598-022-11665-z)
Supplement: Supplementary file 1 — Supplementary Information. [file 41598_2022_11665_MOESM1_ESM.pdf]

No-ozone cold plasma can kill oral pathogenic microbes in H_2O_2-dependent and independent manner.

Nam-Sook Park[1], Se-Eun Yun[2], Hyun-Young Lee[1], Hae June Lee[3], Jeong-Hae Choi[1☏*] and Gyoo-Cheon Kim[1,2☏*].

[1] Department of Research and Development, FEAGLE corporations

[2] Department of Oral Anatomy and Cell Biology, School of Dentistry, Pusan National University

[3] Department of Electrical Engineering, Pusan National University

* These authors are contributed equally for this study.

Corresponding authors:

Jeong-Hae Choi, Department of Research and Development, FEAGLE corporations, 70-6, Jeungsan-ro, Mulgeum-eup, Yangsan-si, Gyeongsangnam-do, 50614, South Korea, e-mail: monday27@feagle.co.kr

Gyoo-Cheon Kim, Department of Anatomy and Cell Biology, School of Dentistry, Yangsan Campus of Pusan National University, Beomeo-ri, Mulgeum-eup, Yangsan-si, Gyeongsangnam 626-870, South Korea, e-mail: ki91000m@pusan.ac.kr

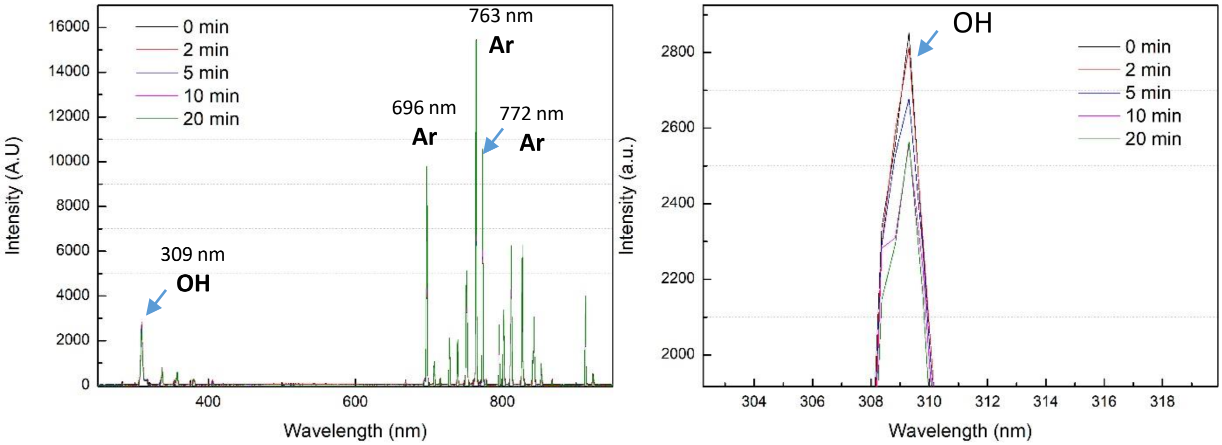

Supplementary Data 1. The time-point OES analysis data of NCP device. OES data was measured at 0, 2, 5, 10 and 20 minutes during total 20 minutes of plasma generation period.

Measuring time	NO (ppm)	NO2 (ppm)
1 min	0.000	0.007
2 min	0.000	0.007
3 min	0.000	0.006
4 min	0.000	0.006
5 min	0.000	0.006
6 min	0.000	0.006
7 min	0.000	0.006
8 min	0.000	0.005
9 min	0.000	0.005
10 min	0.000	0.005

Supplementary Data 2. The results of NO and NO2 measurement from dental-tip mounted NCP device. The sensor of device (MEZUS-210) was placed at 1 cm distance from the end of dental tip.

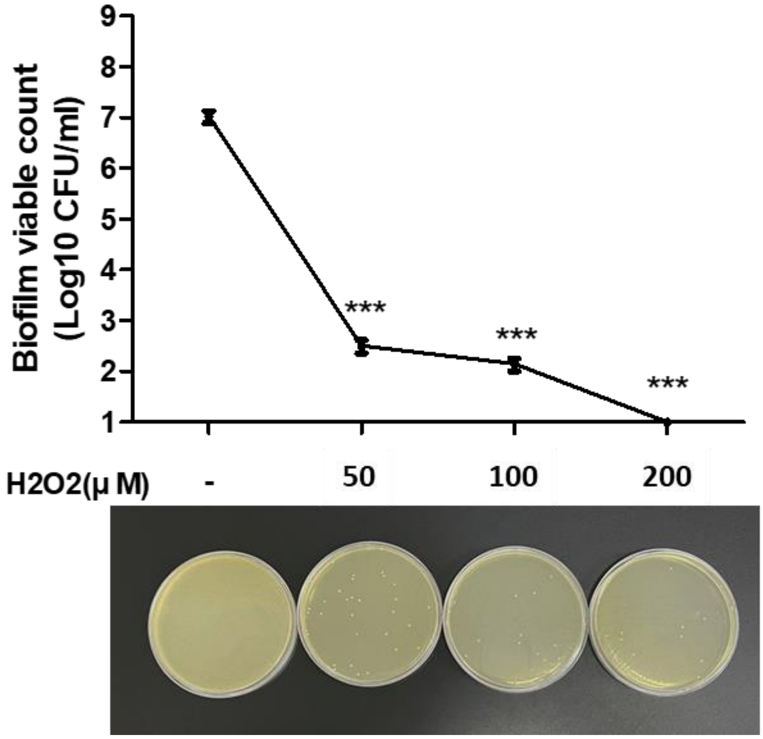

Supplementary Data 3. The effect of H2O2 level in the media on the growth of S.mutans in the liquid media.

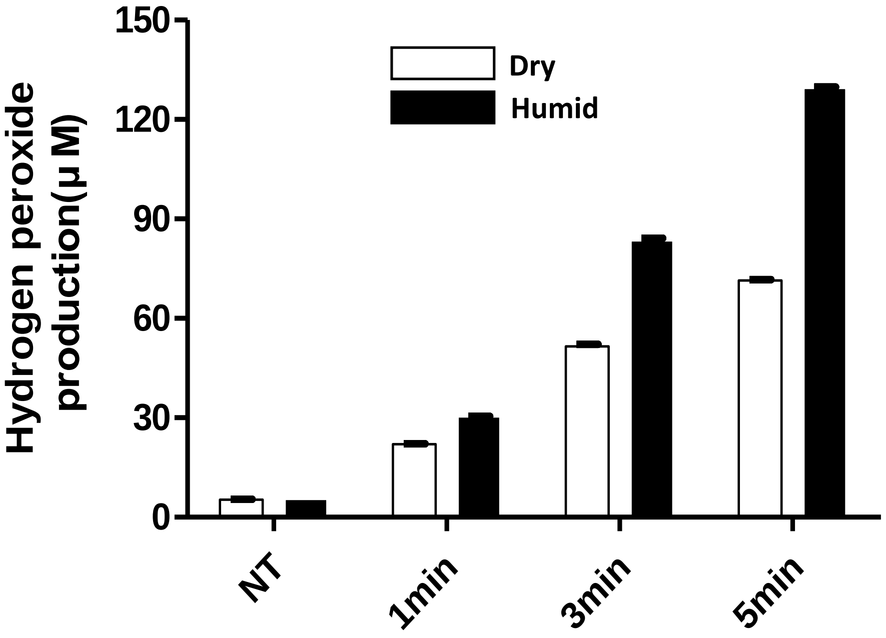

Supplementary Data 4. The effect of the air humidity on the H_2O_2 level in the liquid media. 1 ml of liquid media was subjected to the NCP treatment at dry (32.9%, white bar) or humid (62.4%, black bar) air conditions, and H_2O_2 level was measured.

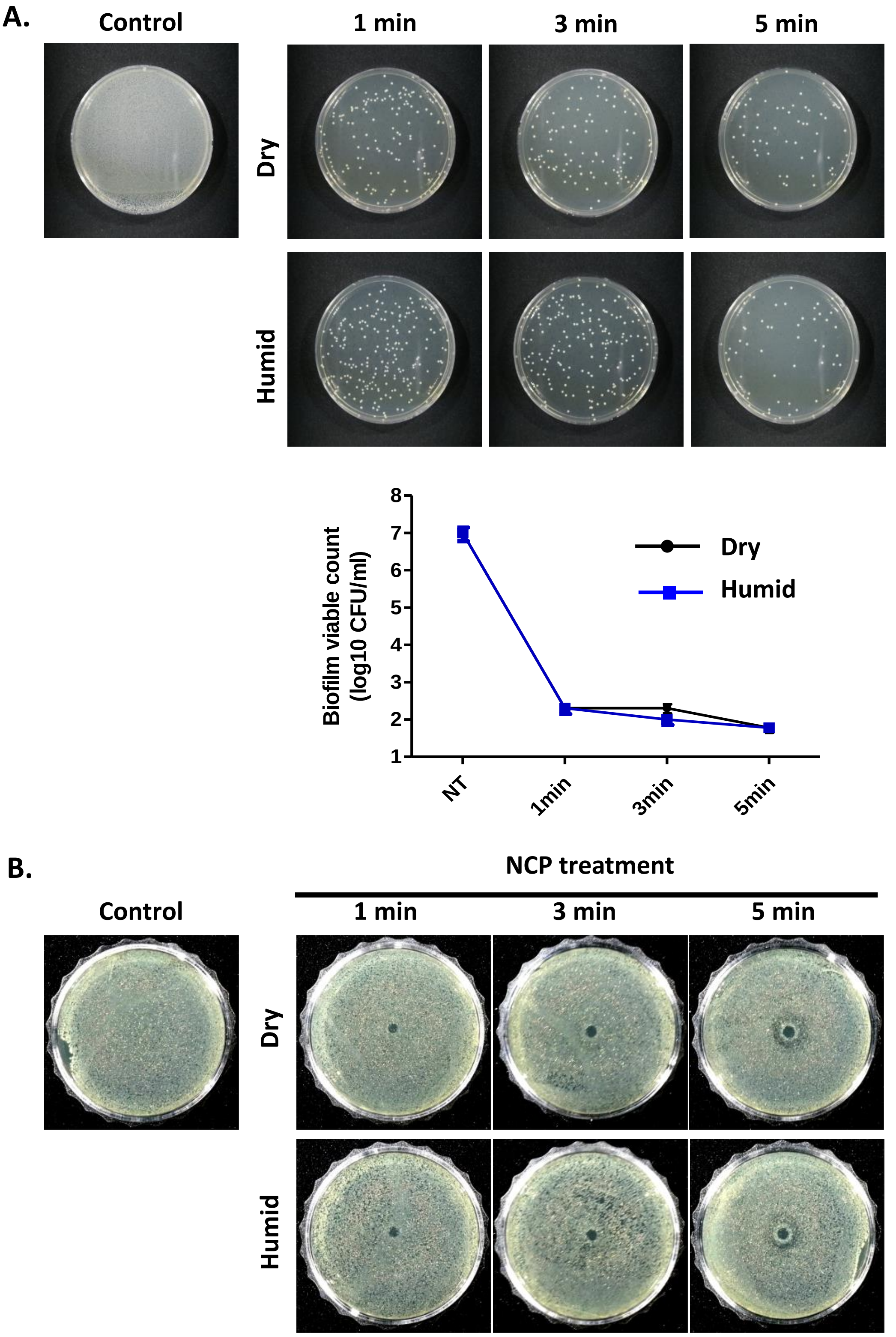

Supplementary Data 5. The effect of the air humidity on the bactericidal activity of NCP. S.mutans cultured at liquid (A) or solid (B) media was subjected to the NCP treatment at dry (32.9%) or humid (62.4%) air conditions.

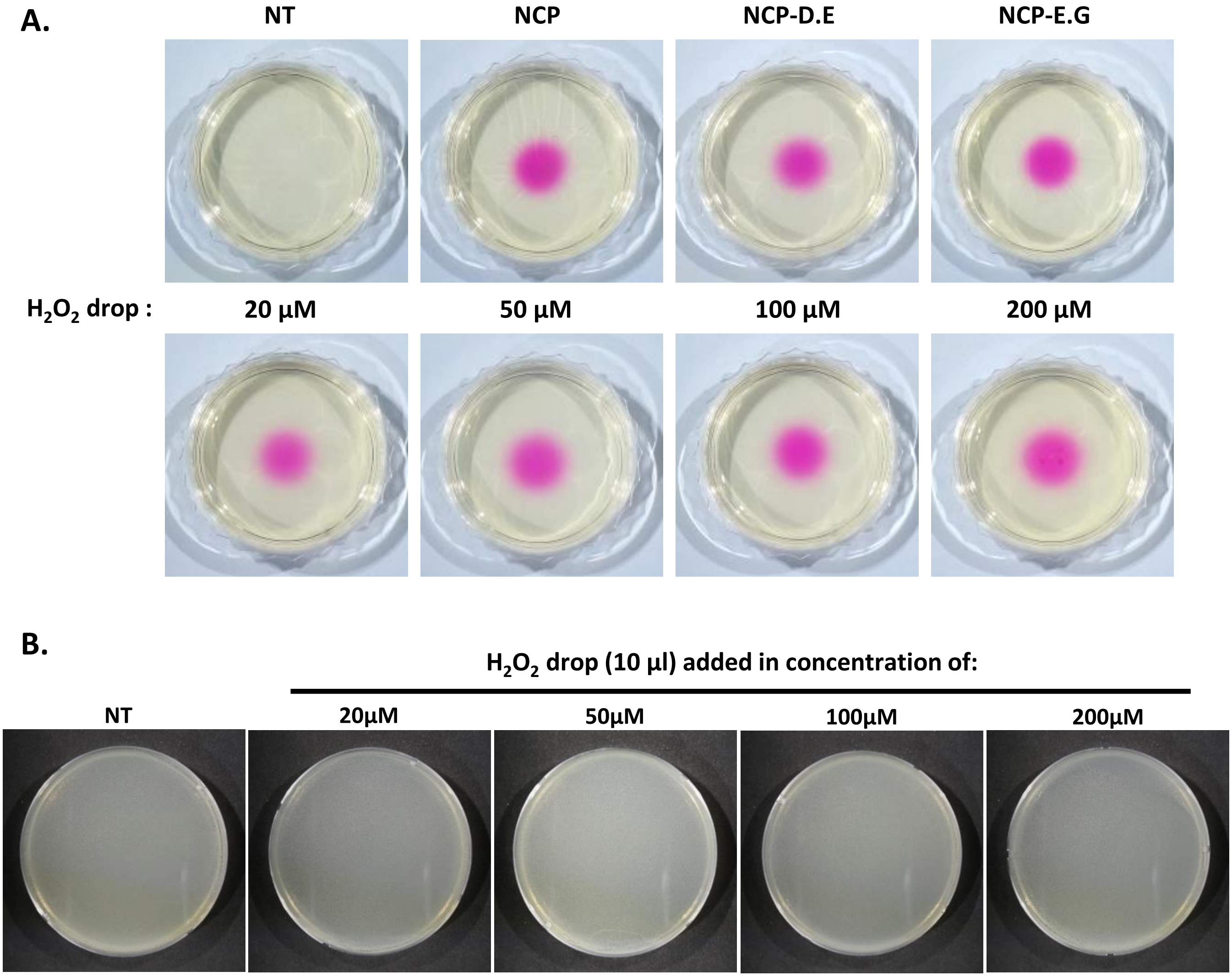

Supplementary Data 6. The antibacterial effect of NCP on solid medium was not dependent of NCP-mediated H_2O_2 generation. (A) The results of modified Amplex-red H2O2 assay showing NCP treatment increased H2O2 level on the surface of solid medium, and this ability of NCP was not affected by electronic grounded mesh (NCP-E.G). For this experiment, a 10 μl of reaction mixture droplet was added on the solid media at the center, and dried for 5 min. Then, the NCP was treated on this solid medium in presence or absence of 2 kinds of meshes for 5 min. (B) The mere addition of a H2O2 droplet (10 μl in concentration of 20, 50, 100 and 200 μM) on S. mutans cultured on solid medium did not created a clear zone.
